# LEAN Methodology to Improve Endoscopy Unit Efficiency in a Multi-subspecialty Ambulatory Surgery Center: A Pilot Study

**DOI:** 10.7759/cureus.69447

**Published:** 2024-09-15

**Authors:** Trilokesh D Kidambi, Harry Trieu, Brian Lilienstein, Peter Hirsch, Charles Erwing, Michael J Sullivan, Lukejohn W Day, Michael W Lew

**Affiliations:** 1 Department of Medicine, Division of Gastroenterology, City of Hope National Medical Center, Duarte, USA; 2 Internal Medicine, University of Southern California Keck School of Medicine, Los Angeles, USA; 3 Anesthesiology, Rosalind Franklin University Chicago Medical School, Chicago, USA; 4 Department of Nursing, Perioperative Service, City of Hope National Medical Center, Duarte, USA; 5 Department of Information Technology, City of Hope National Medical Center, Duarte, USA; 6 Department of Anesthesiology and Perioperative Medicine, City of Hope National Medical Center, Duarte, USA; 7 Department of Gastroenterology, University of California at San Francisco, San Francisco, USA

**Keywords:** anesthesia for upper and lower gi, gastroenterology and endoscopy, improved efficiency, lean methodology, process & performance improvement

## Abstract

Background and objective

Efforts to improve gastrointestinal (GI) endoscopy unit efficiency may lead to increases in colon cancer screening volumes. LEAN management principles applied to GI endoscopy unit practices may serve as a novel foundation for efficiency improvements. We conducted a pilot study in an outpatient, hospital-based GI endoscopy unit with the goal of improving endoscopy efficiency by using LEAN principles

Methodology

A single endoscopist and anesthesiologist along with the nursing care team implemented changes to their practice based on LEAN principles. Efficiency metrics were tracked before these changes and after to assess for improvements.

Results

We observed statistically significant improvements in waiting room time (13.1 minutes vs. 25.6 minutes, p<0.001), recovery room duration (55.5 minutes vs. 61.8 minutes, p=0.01), total facility time (172.5 minutes vs. 196.1 minutes, p<0.001), and true completion time (19.7 minutes vs. 32.3 minutes, p=0.002) after the implementation of LEAN interventions.

Conclusions

A systematic and standardized approach using LEAN methodology can improve GI endoscopy unit operational efficiency. Larger studies are needed to validate our findings and generalize the results to the field broadly.

## Introduction

Colorectal cancer (CRC) is the second leading cause of cancer death in the United States (US) [1], and screening average-risk asymptomatic patients can reduce this risk by enabling early detection and prevention [2,3]. The coronavirus disease 2019 (COVID-19) pandemic caused several delays in cancer diagnosis and treatment [1] and has been associated with a predicted increase in the rate of excess deaths from CRC [4]. This predicted increase has been partly attributed to a decrease in screening colonoscopy volume early in the COVID-19 pandemic [5,6]. As such, efforts to improve efficiency in the gastrointestinal (GI) endoscopy unit can potentially increase screening colonoscopy throughput to meet the expected increase in demand for screening in the US.

Improving efficiency in the endoscopy unit has been studied. Process improvement is predicated on optimizing personnel utilization, room turnover, and recovery time, among other factors [7]. LEAN methodology focuses on the reduction of waste to improve efficiency. Six Sigma focuses on improving quality through consistent practices. Quality control is inherent to the medical field, due to regulations employing LEAN practices that are consistent with improving workflow. LEAN management principles were designed to optimize the manufacturing process by studying the flow of activity and implementing changes to minimize waste and non-value-added activity [8,9].

A previous successful quality improvement (QI) project that applied LEAN management principles in a dedicated GI endoscopy unit at a tertiary care academic center has been shown to improve efficiency metrics and save costs [8,9]. Recently, utilization of the Plan-Do-Study-Act (PDSA) QI methodology has been shown to be effective in assessing operational metrics and identifying opportunities for improvement in an endoscopy unit [10], and simply shifting responsibility to patient transport into the room to the GI team has been shown to improve turnover time [11].

Previous efficiency-related research has so far focused on dedicated endoscopy units [12,13,14,15], but there are no published studies on GI endoscopy unit efficiency in a multi-specialty, hospital-based ambulatory surgery center setting. In light of this, we conducted this study to (1) measure baseline operation efficiency metrics in a GI endoscopy unit within a hospital-based, ambulatory surgery center; (2) apply LEAN methodology with the intent of improving efficiency; and (3) re-measure operational efficiency metrics after LEAN QI interventions to assess for improvement compared to baseline.

## Materials and methods

Study setting

The study was performed in the outpatient GI endoscopy unit at the City of Hope (Duarte, CA), a National Comprehensive Cancer Network-designated tertiary cancer center in Southern California. The outpatient GI endoscopy unit consists of a single dedicated daily procedure room within a hospital-based, ambulatory surgery center comprising four outpatient operating rooms and four minor procedure rooms; a second minor procedure room is used for outpatient GI procedures on an ad hoc basis.

GI endoscopy procedures are performed by board-certified attending gastroenterologists. Esophagogastroduodenoscopy (EGD) and colonoscopy are the primary procedures; one gastroenterologist also performs endoscopic ultrasound (EUS) procedures in this unit. Sedation is provided by board-certified attending anesthesiologists and/or medically directed certified registered nurse (RN) anesthetists; most procedures are performed under monitored anesthesia care (MAC). Four gastroenterologists rotate (one gastroenterologist per day) and perform 1,600 procedures per year in the outpatient GI unit. All surgeons and proceduralists of the ambulatory surgery center share the registration, preoperative and postoperative areas and staff, transport personnel, and environmental services. However, staffing solely dedicated to GI endoscopy is the intraoperative team of nursing and endoscopy technicians and a sterile processing technician.

The GI endoscopy team comprises the endoscopist (gastroenterologist), an anesthesiologist, one or two endoscopy-trained RNs, one or two endoscopy-trained technicians, and a designated technician to disinfect and clean the endoscopes. Staffing of one or two RNs and technicians is predicated on staffing assignments. When two RNs and/or technicians are assigned, they alternate procedures and take breaks when not in the room. A “floating” RN or technician provides breaks when only one RN or technician is assigned.

There are 11 beds in the preoperative bay and 11 beds in the postoperative bay, which are allocated to the ambulatory surgery center (i.e., endoscopy shares the preop and postop areas with surgery). In the post-anesthesia care unit (PACU), the nurse ratio is 1:2 for GI patients.

Study design

This study received approval from the City of Hope Institutional Review Board (COH Protocol #/Ref#: 22234/225664). After obtaining approval, a prospective, pilot study design was implemented to assess improvements in GI endoscopy efficiency by utilizing LEAN methodology. We included outpatient GI endoscopy procedures performed by a single endoscopist (TDK), who performed only general endoscopy procedures at the outpatient center and agreed to participate in the study. The anesthesiologist was assigned the entire lineup for the day. The anesthetic plan was left to the discretion of the anesthesiologist. We included only a single anesthesiologist (MWL) who agreed to participate in the study.

This study consisted of three parts. The first part was to create a diagram of the current state workflow and collect data regarding the existing process. The second part involved gathering input and collaboration from the endoscopy and perioperative teams and an electronic health record consultant. The third part constituted the implementation of LEAN principles and the evaluation of the impact on various operational metrics. The goal of this study was to discuss simplified LEAN improvements [16], which were free and involved the use of already allocated resources.

Data collection

Endoscopy volume data were gathered throughout the study period. Additionally, established [7] GI endoscopy efficiency metrics were collected, such as on-time first start, on-time case start, waiting room time, pre-procedure time, room duration (wheels-in to wheels-out), turnover time (wheels-out to wheels-in), recovery room time, and total time in the facility. We also calculated the true completion time [8].

Statistical analysis

Procedures performed in 2021 or 2022 were assigned to the pre-LEAN improvements group, and those performed in 2023 were assigned to the post-LEAN improvements group. Continuous variables in the two groups were compared using a two-sample t-test, while categorical variables were compared using Pearson’s chi-squared test and Fisher’s exact test. Statistical analyses were performed using Stata/BE 18.0 (StataCorp LLC, College Station, TX).

## Results

Examining workflows and developing/implementing new processes: first and second parts

**Figure 1:**
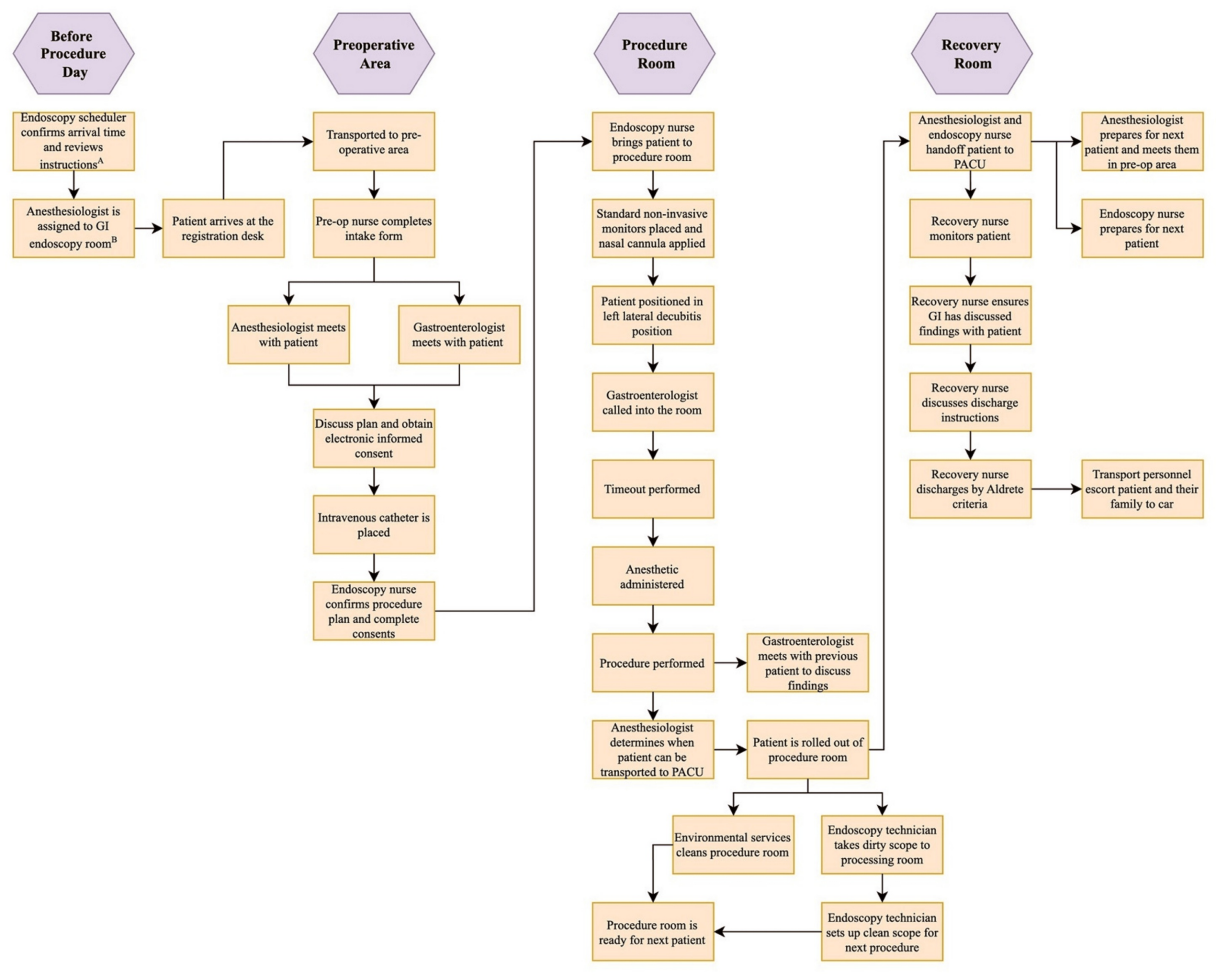
Outpatient gastrointestinal endoscopy workflow ^A^Performed one week prior to the procedure. ^B^Assigned at 6 pm the evening prior to the procedure based on scheduled call positions and not by task or service-specific talents

The outpatient GI endoscopy workflow was examined in detail and is depicted in the flowchart shown in Figure 1. LEAN principles [16], with the primary goal of eliminating waste to improve efficiency without incurring increased resources or costs, were applied to understand areas for improvement in workflow analysis as shown in Table 1. All GI endoscopy team members and perioperative teams participated in providing recommendations for operational improvement. These frontline workers were considered the experts in their area given their extensive understanding of the processes. Additionally, a specialist focused on optimizing the electronic health record documentation observed and recommended workflows to enhance the efficiency of documentation and charting. All feedback was then discussed with a specialist (Lean Six Sigma Black Belt) in LEAN workflows (MWL) and a standardized workflow was implemented by a subgroup (TDK and MWL) with an emphasis on their workflows as summarized in Table 2.

**Table 1:** Lean wastes and proposed interventions ^1^Improve patient flow, supplies, and equipment. ^2^Reduce waste of motion. ^3^Redundancies, creating too much, duplication of tests. ^4^Unnecessary tests, different forms with same information, repetitive processes EMR: electronic medical record; PACU: post-anesthesia care unit

Lean wastes	Our interventions
Reduce wait/idle time	Patient arrival time minimized
	Work in parallel and not series
	Communication of equipment needs
	Preround patients
Minimize inventory	Consistency in practice (use the same disposables in every case)
	No propofol pump tubing, use only 20 ml vs. 50 ml propofol bottles
	Hand vs. infusion pumps
Eradicate defects to improve quality of care	Constant review of system/practice patterns to be efficient
	Refine practice
	Prerounding of charts the night before
	Complete EMR after each case
Transportation^1^	Stocked shelves in the room to not waste time out of the room
	Pre to intra to postop is a one-way pattern flow
	Drop patient off in PACU, see next patient in preop, then go to the procedure room
Prevent injuries^2^	Patient positions self for comfort and eliminate positioning injuries (staff safety)
	The patient recovers in lateral position and returns to supine by him/herself
Minimize overproduction^3^	Case times are not scheduled excessively to avoid gaps yet reduce wait time
	Use of EPIC templates
	Blood glucose one-time check
	Staff trained to mobilize/encourage patients to be ready for discharge
Eliminate overprocessing^4^	GI order set for PACU anesthesia
Untapped human potential	Once wastes are minimized/eliminated staff morale and commitment increase
	Additional cases that increase revenue

**Table 2:** Summary of efficiency metric interventions by role WI-WO: walk in-walk out

Efficiency metric	Interventions		
	Endoscopist	Anesthesiologist	Peri/intraoperative nursing
On-time start	Prefilling electronic consents; utilization of templates, consenting patients in advance of procedure during room turnovers	Reviewing case lists prior to the day of procedures, pre-populating templates, getting consent from patients in advance of procedure during room turnovers	
On-time start for the first case of the day	Arriving 15-30 minutes prior to the first case of the day	Arriving 15-30 minutes prior to the first case of the day	
Pre-procedure time	Prefilling electronic consents; utilizing templates, getting consent from patients in advance of procedure during room turnovers	Reviewing case lists prior to the day of procedures, pre-populating templates, consenting patients in advance of procedure during room turnovers	Witnessing and completing electronic consents, placing monitors in the preoperative unit
Room duration (WI-WO)	Entering room as patient wheeled in, avoiding repositioning for combined EGD/colonoscopy procedures, providing an estimate of case duration intra-procedure ("5 minute-warning")	Titrating sedation based on case duration	Initiating blood pressure measurement once in the room, arranging postoperative unit space based on case duration estimate
Recovery room duration	Providing an estimate of case duration	Propofol alone when feasible; titrating sedation based on case duration provided by endoscopist; evaluating patients for discharge during room turnovers	Active assessment of Aldrete score; family member/patient's ride brought into postoperative unit early
Turnover time	Providing the technician with a list of anticipated equipment in advance		
In facility total duration	Completing note and discharge paperwork immediately after the procedure; providing results to family members in the waiting area immediately after the procedure	Evaluating patients for discharge during room turnovers	Family member/patient's ride brought into the postoperative unit early
True completion time			Calling the last patients of the day sequentially to fill gaps created by same-day cancellations

Volume and operational endoscopy efficiency metrics were gathered between January 1, 2021, and December 31, 2022, to serve as the baseline against which comparisons would be made after implementing LEAN methods. The baseline data are presented in Tables 3-4.

**Table 3:** Summary of procedures and volume performed EGD: esophagogastroduodenoscopy

Procedures and volume performed			
Number and types of procedures performed			
	2021	2022	2023
Number of days of pilot	16	13	14
Number of first cases	14	12	11
Total procedures performed, n	195	150	141
Colonoscopies, n	129	87	85
EGDs, n	59	61	52
Flexible sigmoidoscopies, n	7	2	4

**Table 4:** Summary of the impact of the interventions ^1^Mean time in minutes. ^2^Continuous variables were compared using a two-sample t-test, while categorical variables were compared using Pearson’s chi-squared and Fisher’s exact tests. ^3^On-time start was calculated based on the number of patients undergoing procedures, not based on the number of procedures. If a patient was having two procedures, such as an EGD and colonoscopy, they would only be counted once towards the on-time start of the first procedure performed. Hence, there is an expected difference between number of procedures performed and on-time starts. *p<0.05 SD: standard deviation; WI-WO: walk in-walk out

Impact of interventions on efficiency metrics			
	Pre-intervention	Post-intervention	Statistical significance (p-value)^2^
On-time start^3^,n (%)	127/274 (46.4%)	79/113 (69.9%)	<0.0001*
On-time start if first case, n (%)	24/26 (92.3%)	10/11 (90.9%)	1.000
Waiting room time, minutes, mean ± SD^1^	25.6 ± 21.2	13.1 ± 14.9	<0.0001*
Pre-procedure time, minutes, mean ± SD^1^	77.4 ± 29.4	71.6 ± 27.2	0.0696
Room duration, WI-WO, minutes, mean ± SD^1^	26.5 ± 9.8	27.3 ± 10.5	0.4974
Recovery room duration, minutes, mean ± SD^1^	61.8 ± 23.6	55.5 ± 20.4	0.0140*
Turnover time, minutes, mean ± SD^1^	16.6 ± 7.9	16.4 ± 8.3	0.8423
In-facility total duration, minutes, mean ± SD^1^	196.1 ± 41.7	172.5 ± 38.7	<0.0001*

Implementing LEAN methods: the third part

The planned and implemented workflow adjustments were categorized as those performed by the endoscopist, the anesthesiologist, and the assigned nursing team and are summarized in Tables 1-2. The majority of the changes were implemented by the anesthesiologist and endoscopists and these included arriving early and optimizing activities during room turnovers.

Volume data and operations metrics during the LEAN implementation phase were gathered between January 1, 2023, and December 31, 2023, and shown in Tables 3-4. There were statistically significant improvements in waiting room time (13.1 minutes vs. 25.6 minutes, p<0.001), recovery room duration (55.5 minutes vs. 61.8 minutes, p=0.01), total facility time (172.5 minutes vs. 196.1 minutes, p<0.001), and true completion time (19.7 minutes vs. 32.3 minutes, p=0.002) after the implementation of LEAN interventions.

## Discussion

In this pilot study conducted at an endoscopy practice within a multi-subspecialty, hospital-based ambulatory surgical center, we successfully applied the LEAN methodology to improve operational efficiency. By implementing no-cost interventions, and utilizing already available resources, we reduced total facility time by improving waiting room time and recovery room duration. Furthermore, we observed an improvement in true completion time by greater than 10 minutes. Our findings align with recent studies of endoscopy unit efficiency, including those employing simulation [7] and real-world data [8,10,11]. Similar to Kaushal [8] et al., we used efficiency analysis as the foundation for our QI interventions and demonstrated that operational metrics could be improved in the endoscopy unit. By applying the LEAN methodology to our specific endoscopy unit practice, we improved operational metrics including true completion time [8], an operational benchmark that represents how suboptimal efficiency can have adverse effects propagated throughout an entire day, rather than simply on a case-by-case basis.

Additionally, while our study's improvements in operational metrics are similar to those of Bradley et al. [10], we showed that a “ramp” model inherent to the PDSA QI framework is not necessarily required to gain stakeholder buy-in. We implemented several agreed-upon LEAN interventions at once rather than sequentially as in a previous study, and this was effective. However, we limited our interventions to a pilot study with a single endoscopist and anesthesiologist with the intention of showing “proof of concept” before working toward a more generalized implementation.

A recent study by Post et al. [11] specifically focused attention and interventions on turnover time in the endoscopy unit and found a single intervention of switching responsibility for patient transport into the room to the GI team improved the metric. Our GI team is already responsible for patient transport into the endoscopy suite, and hence we were unable to utilize this intervention. Also, while several of our interventions were meant to improve turnover time, we did not see such an improvement, which may have been due to sample size or already optimized turnover time at baseline. However, we hypothesize that by completing several tasks during the room turnover, we reduced time in the post-procedure recovery unit, which contributed to the overall decrease in total facility time. Furthermore, the “five-minute warning” communicated by the endoscopist to the anesthesiologist likely allowed for appropriate medication titration that reduced recovery room time.

There are several limitations to our study. This constituted a pilot study with a small sample size due to the inclusion of only a single endoscopist and anesthesiologist, given limited interest from other providers. However, the processes used and the specific interventions can be applied internally in our practice setting among additional endoscopists and anesthesiologists and more broadly to other similar practice settings. Additionally, as all participants were aware of the pilot, the Hawthorne effect could have impacted results positively.

## Conclusions

The primary finding of our study is that a systematic and standardized approach using LEAN methodology to improve endoscopy unit operational efficiency is achievable. We believe that the specific interventions we implemented are less notable than the process used to arrive at them. Thus, we intend to report the results to promote the application of our findings at our institution and provide a framework for adoption among other similar endoscopy units.
